# Distinct Human Stem Cell Populations in Small and Large Intestine

**DOI:** 10.1371/journal.pone.0118792

**Published:** 2015-03-09

**Authors:** Julie M. Cramer, Timothy Thompson, Albert Geskin, William LaFramboise, Eric Lagasse

**Affiliations:** 1 Department of Pathology, University of Pittsburgh School of Medicine, 200 Lothrop Street, Pittsburgh, PA, 15261, United States of America; 2 McGowan Institute for Regenerative Medicine, University of Pittsburgh, 450 Technology Drive, Suite 300, Pittsburgh, PA, 15219, United States of America; 3 Department of Bioengineering, University of Pittsburgh, 3700 O'Hara St, Pittsburgh, PA, 15261, United States of America; 4 Department of Pathology, University of Pittsburgh, Shadyside Hospital, West Wing, WG 02.11, 5230 Center Avenue, Pittsburgh, PA 15232, United States of America; Rush University Medical Center, UNITED STATES

## Abstract

The intestine is composed of an epithelial layer containing rapidly proliferating cells that mature into two regions, the small and the large intestine. Although previous studies have identified stem cells as the cell-of-origin for intestinal epithelial cells, no studies have directly compared stem cells derived from these anatomically distinct regions. Here, we examine intrinsic differences between primary epithelial cells isolated from human fetal small and large intestine, after *in vitro* expansion, using the Wnt agonist R-spondin 2. We utilized flow cytometry, fluorescence-activated cell sorting, gene expression analysis and a three-dimensional *in vitro* differentiation assay to characterize their stem cell properties. We identified stem cell markers that separate subpopulations of colony-forming cells in the small and large intestine and revealed important differences in differentiation, proliferation and disease pathways using gene expression analysis. Single cells from small and large intestine cultures formed organoids that reflect the distinct cellular hierarchy found *in vivo* and respond differently to identical exogenous cues. Our characterization identified numerous differences between small and large intestine epithelial stem cells suggesting possible connections to intestinal disease.

## Introduction

The intestine consists of two major subdivisions: the small intestine (SI) and the large intestine (LI), which differ in structure and function. The SI is largely responsible for the digestion and absorption of food while the LI aids in final water absorption and waste removal. Among other signaling pathways, Wnt and Notch control the well-defined epithelial hierarchy in the intestine, helping to maintain stem cell homeostasis. Since these pathways require receptors, ligands and transcriptional regulation, it is unclear whether differences observed between the SI and LI are primarily due to intrinsic or extrinsic mechanisms [1,2]. Understanding these differences is crucial, since failure of intestinal stem cells to properly proliferate and differentiate may lead to cancer, which is 20 times more prevalent in the LI than the SI in humans [3]. However, a thorough investigation of the origin of the differences between the SI and LI has yet to be done.

The identification and characterization of stem cells in the intestine has evolved rather rapidly in recent years. *In vivo* lineage tracing studies have identified leucine-rich repeat-containing G-protein coupled receptor 5 (LGR5)^+^ stem cells in the mouse as cells capable of generating all the epithelial cells of the intestine and forming crypt-like structures *in vitro* [4,5]. Interestingly, LGR5 is intricately involved in the synergistic activation of the Wnt pathway, via the R-Spondin protein family, which is responsible for homeostatic crypt formation and maintenance in the intestine [6–8]. This pathway is also commonly altered in colon cancer via mutation of adenomatous polyposis coli (APC), causing an accumulation of beta-catenin in the nucleus and enhanced Wnt signaling [9,10]. Rapidly growing adenomas form in the mouse after deletion of APC in LGR5^+^ intestinal stem cells, suggesting that normal stem cells are the cell-of-origin of intestinal cancer [11]. Additionally, murine adenomas revealed continual LGR5^+^ stem cell activity, providing functional evidence of a cancerous stem cell population in primary intestinal adenomas [12].

The Wnt pathway has extensive cross-talk with the Notch pathway in its control over cell fate decisions, proliferation and tumorigenesis [1,13,14]. More specifically, activation of the Notch pathway represses secretory cell differentiation but inhibition of the Notch pathway leads to activation of atonal homolog 1 (ATOH1) promoting goblet cell differentiation (S1 Fig.) [1,15,16]. Thus far, a majority of studies elucidating these pathways in the intestine have not made clear distinctions between the SI and LI, possibly missing differences with important consequences.

The majority of intestinal stem cell characterization has been performed in animal models because cells from normal human intestine has been notoriously difficult to grow *in vitro* and lineage tracing cannot be performed practically in humans. To overcome these limitations, we used feeder cells as a stromal layer to provide cell-cell interactions with human intestinal cells and promote epithelial cell growth [17]. Our lab has previously used this system to isolate and expand tumor cells with stem cell properties (cancer stem cells, CSCs) from human metastatic colon cancer [18]. Here, we isolated human fetal intestinal cells from primary tissue and expanded the cells on the feeder layer. Other *in vitro* models have successfully been used to study and understand stem cell biology such as the three-dimensional system introduced by Sato, et al [5]. Importantly, we compared cells expanded from SI and LI isolated from the same donor tissue, minimizing potential discrepancies due to genetic variability. We also expanded the SI and LI cells in identical culture conditions, to allow for the comparison of intrinsically programmed differences. The stem cell enriched cultures from human SI and LI allowed us to compare stem cell and differentiation properties to determine key differences between these two cell populations, with possible implications to disease occurrence in these two regions of the intestine.

## Results

### SI and LI epithelial cells expanded in vitro are enriched for cells expressing stem cell markers

**Fig 1:**
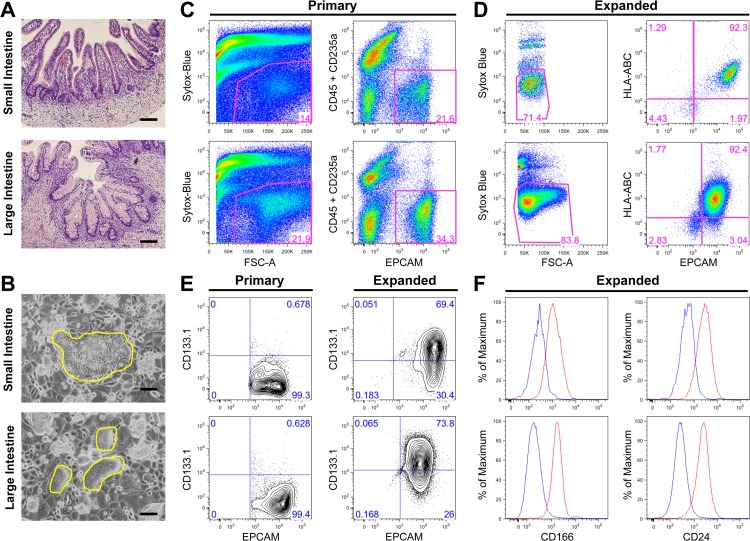
*In vitro* expanded primary epithelial cells from human fetal small and large intestine express stem cell markers. **A-F**, top panels: small intestine, bottom panels: large intestine (**A**) Hematoxylin and eosin paraffin sections of normal fetal intestine at 23 weeks of gestation. (**B**) Intestinal cells expanded on the feeder layer (yellow outline). (**C-F**) Representative analyses of primary and expanded cells at passages 4, 5 or 6 with the percentage of gated live cells provided. (**C**) Flow cytometric analysis (dot plots) of freshly isolated primary intestinal cells. Gating strategy identifies total live cells (left panels) and EPCAM^+^ cells, excluding blood cells (CD45^+^CD235a^+^) (right panels). (**D**) Flow cytometric analysis of expanded cells. Gating strategy identifies total live cells (left panels) and EPCAM^+^ cells, excluding rodent feeder layer cells (HLA^−^) (right panels). (**E**) Flow cytometric analysis (5% probability contour plots) of primary and expanded intestinal cells, stained with EPCAM and CD133.1. (**F**) Expanded intestinal cells also express other putative stem cell markers. Histograms represent unstained control (blue) and stained with CD166 or CD24 (red) of live EPCAM^+^ cells. Scale bars = 100μm for panel A and B.

To investigate the *in vitro* potential of cells isolated from the SI and LI, we used human intestinal tissues around gestational week 23. At this stage, crypts in both fetal SI and LI are structurally similar to those observed in adult, consisting of epithelial cells of all lineages, with the exception of a higher number of goblet cells in fetal tissue (Fig. 1A). We used a feeder layer to expand cells from the SI and LI as it has previously been shown to expand human colon CSCs from metastatic colon cancer specimens [18]. In addition, we found that growth of normal fetal intestinal cells on the feeder layer required the addition of R-spondin 2, a Wnt agonist acting via the LGR5 receptor [8,19]. This was not surprising considering that R-spondin 1 is known to be important for the *in vitro* expansion of intestinal cells and all four R-spondins bind the same receptor family and are expected to be somewhat redundant [5,8]. In total, 29 pairs of SI and LI were isolated and processed for culture on the feeder layer. After plating ∼80,000 primary cells/cm^2^, only 18 pairs formed rare colonies after approximately two weeks (62% success rate), indicating that only a small population of cells from primary tissue was capable of surviving selection under these culture conditions. Some samples were much more efficient at forming colonies than others, resulting in an estimated efficiency rate range of 0.00015–0.005%. Serial passaging led to more robust expansion of intestinal cells. Phenotypic differences in colony density, cell size and shape were observed between cells expanded from SI and LI and could be passaged at least 10 times with no apparent morphological changes or oncogenic transformation (Fig. 1B and S1 Table). After primary cell expansion *in vitro*, flow cytometric analyses revealed enrichment of epithelial cells, as marked by Epithelial Cell Adhesion Molecule (EPCAM), from 21.6 to 92.3% in cells expanded from SI and from 34.3% to 92.4% in cells expanded from LI (Fig. 1C&D). Over twenty cell-surface markers were interrogated for all SI and LI epithelial cell cultures. The most reproducible and relevant markers indicated that expanded intestinal cells expressed several markers associated with stemness. CD133.1 (AC133) has been suggested as a marker of CSCs in the intestine [20–22], as well as a marker for normal stem cells such as hematopoietic stem cells [23] or neuronal stem cells [24]. While primary cells from SI and LI contained less than 1% CD133.1^+^ cells, approximately 70% of the expanded cells expressed CD133.1 (Fig. 1E). Other putative stem cell markers showed similar expression profiles, including CD166 [25] and CD24 [26], both enriched in expression over primary cells, similar to CD133.1 (Fig. 1F and data not shown) [18]. A previous study using human tissue from three months to eighteen years of age reported isolation, characterization and expansion of stem cell populations from single cells [27]. Attempts were made to expand fetal primary intestinal cells after cell sorting, but no robust long-term colonies were generated from sorted primary cells. The developmental difference in pre-natal versus post-natal and adult tissues may account for our failure to generate long-term colonies from single cells. The environment is much more dynamic and mucinous in the prenatal developmental state even though adult-like structures are present. We therefore expanded bulk intestinal epithelial cells *in vitro* directly from dissociated tissue prior to further characterization.

### Differential expression of stem cell surface markers separate disparate colony-forming activity in cells expanded from SI and LI

**Fig 2:**
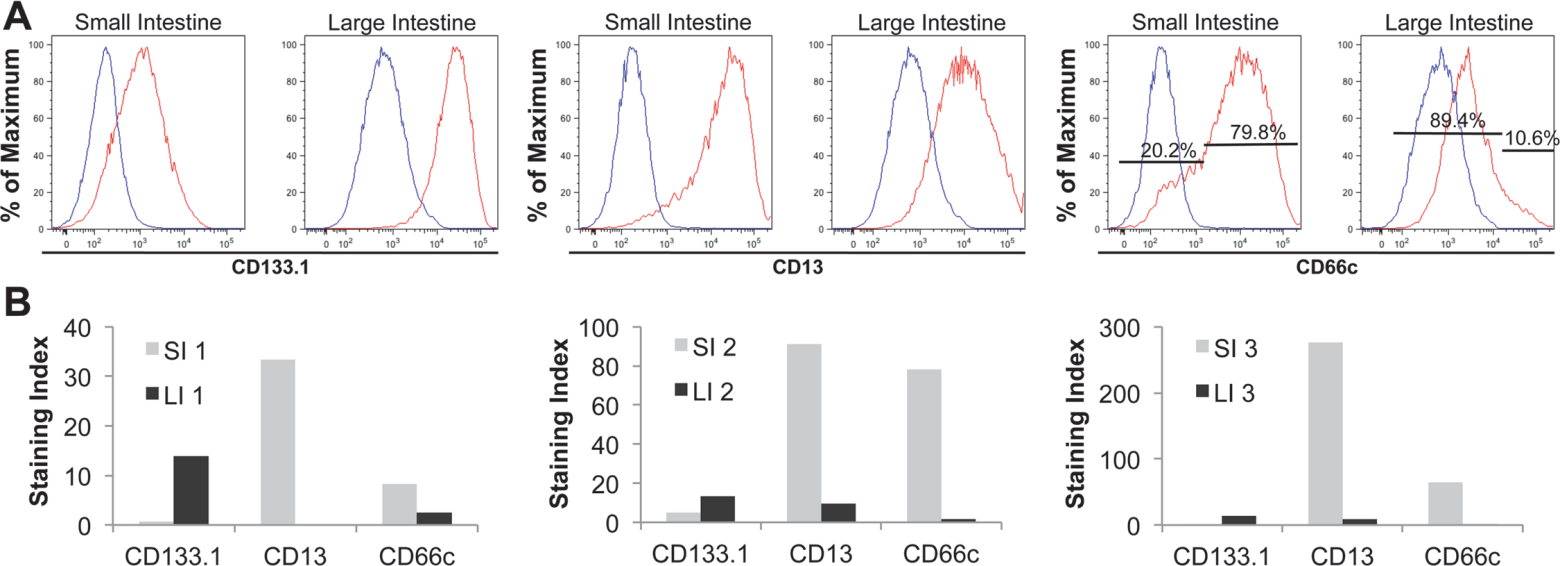
Expanded small and large intestinal cells show different cell surface marker expression. (**A**) Flow cytometric analysis of CD133.1, CD13 and CD66c expression on expanded cells from one paired SI and LI sample (SI and LI depicted here originated from the same tissue source). The histograms show one representative set of three paired samples examined presenting unstained control (blue) and stained live, EPCAM^+^ cells (red). For CD66c, the percentage of low and high expressing cells is provided. All markers were PE-labeled. The same control was used for SI CD13 and CD66c as well as LI CD133 and CD13 because the staining was done at the same time. (**B**) Corresponding staining index of three paired samples showing reproducible results between the paired SI and LI samples (1, 2 and 3) (staining index = D/W, where D is the distance between the positive and negative populations and W is two standard deviations of the negative population).

We observed greater expression of CD133.1 in cells expanded from LI compared to SI, while CD13, an aminopeptidase responsible for hydrolyzing N-terminal amino acids at the brush border membrane, showed higher expression in the SI (Fig. 2A). [28]. In addition, CD66c, also known as carcinoembryonic antigen-related cell adhesion molecule 6 (CEACAM6), was notably higher in cells expanded from SI (79.8% CD66c^hi^) compared to cells expanded from LI (89.4% CD66c^low^) (Fig. 2A). These trends were consistent in expanded cells from three paired SI and LI samples as seen by staining index profiles (Fig. 2B). The staining index describes the extent to which a positive population is resolved from the negative control population.

To demonstrate the functional importance of the differential expression of cell surface markers, we used limiting dilution analysis (LDA) to assess the clonogenic potential of epithelial cell populations derived from SI and LI. LDA is a quantitative measurement that can determine the colony-forming frequency of a specific cell population. Increased colony-forming frequency correlates with stem cell activity [29]. Since CD66c showed a heterogeneous and opposite expression pattern in cells expanded from SI and LI, we asked whether it could distinguish colony-forming ability within these two populations of intestinal epithelial cells. Using Fluorescence-Activated Cell Sorting (FACS), we sorted expanded cells from three tissue-matched samples of SI and LI in limiting dilution based on high and low CD66c expression. Four weeks after plating, we calculated the colony-forming unit (CFU) frequency of the cells by linear regression [30]. The frequency of the EPCAM^+^ colony-forming cells from SI and LI was similar, and ranged from 1/10 to 1/106. Interestingly, cells expanded from SI that expressed low levels of CD66c had a higher CFU frequency than CD66c^hi^ cells, while LDA results for cells expanded from LI were reversed (Table 1). Therefore, even though a majority of cells expanded from SI are CD66c^hi^, as seen in the staining index (Fig. 2B), the subpopulation of CD66c^low^ cells are the cells with more colony-forming ability (Table 1). The opposite is true for cells derived from LI. In consequence, the disparity in expression of CD66c can be used to enrich for expanded SI and LI cells with self-renewal potential.

**Table 1:** Limiting Dilution Analysis (LDA) indicates that opposing CD66c expression separates colony-forming ability in epithelial cells expanded from SI and LI.

Sample	Markers	CFU Freq.	SE Range in CFU Frequency
**SI 1**	HLA+/EpCAM+	1/60	1/73–1/49
	HLA+/EpCAM+/CD66c^low^	1/11	1/15–1/8
	HLA+/EpCAM+/CD66c^hi^	1/78	1/103–1/58
**SI 2**	HLA+/EpCAM+	1/10	1/16–1/6
	HLA+/EpCAM+/CD66c^low^	1/12	1/17–1/9
	HLA+/EpCAM+/CD66c^hi^	1/25	1/30–1/18
**SI 3**	HLA+/EpCAM+	1/25	1/30–1/20
	HLA+/EpCAM+/CD66c^low^	1/11	1/16–1/8
	HLA+/EpCAM+/CD66c^hi^	1/34	1/45–1/25
**LI 1**	HLA+/EpCAM+	1/22	1/27–1/18
	HLA+/EpCAM+/CD66c^low^	1/24	1/32–1/18
	HLA+/EpCAM+/CD66c^hi^	1/9	1/13–1/7
**LI 2**	HLA+/EpCAM+	1/106	1/129–1/87
	HLA+/EpCAM+/CD66c^low^	1/211	1/279–1/160
	HLA+/EpCAM+/CD66c^hi^	1/15	1/20–1/11
**LI 3**	HLA+/EpCAM+	1/38	1/47–1/31
	HLA+/EpCAM+/CD66c^low^	1/77	1/103–1/58
	HLA+/EpCAM+/CD66c^hi^	1/13	1/18–1/10

### Gene expression analysis of epithelial cells expanded from SI and LI indicated separate molecular signatures and distinct intrinsic regulation

**Fig 3:**
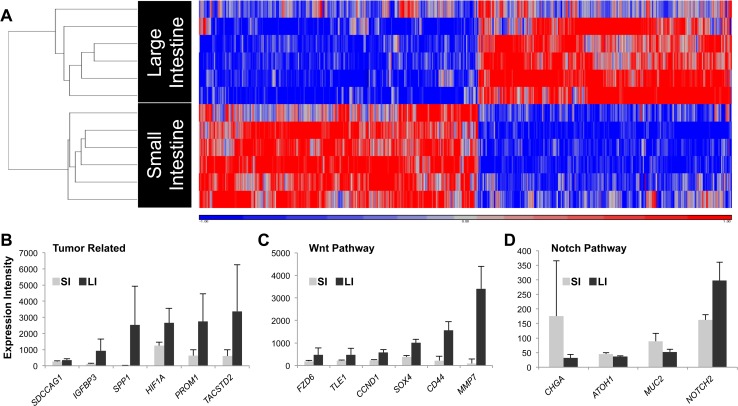
Gene array analysis revealed differing molecular signatures between cells expanded from SI and LI. (**A**) Heat map of differentially expressed transcripts of the expanded epithelial cells from SI versus LI. Unsupervised clustering was performed on log2 gene expression values (K-means algorithm) with q<0.05 (FDR = 5%) cutoff. Expression values are scaled for minimum (blue) and maximum (red) intensity values. Expanded cells formed distinct clusters based on cell origin (SI or LI). (**B-D**) Histograms depict significant (q<0.05) differential expression of individual genes between cells expanded from SI and LI classified into canonical pathways using Ingenuity Pathways Analysis (see Methods). All values are reported as mean (box) plus standard deviation (error bar) of raw intensity for each transcript. List of gene names can be found in S2 Table.

We showed that expanded epithelial cells from SI and LI are enriched for intestinal stem cells as evidenced by their dependence on R-Spondin 2, their expression of stem cell markers (Fig. 1) and their high colony-forming frequency (Table 1). We then used gene array analysis to determine if cells expanded from SI and LI demonstrated differences in their molecular signatures (NCBI Gene Expression Omnibus: GSE56525). Among the 21,238 total probes on each array, 3274 probes were significantly different. After removal of probes for the same gene, 1118 transcripts had an expression fold change greater than 1.5. Cells expanded from SI and LI clustered separately using unsupervised hierarchical clustering methods where the distances are defined linearly (left axis, Fig. 3A). Therefore, even though we were unable to distinguish subregions in SI tissue (ileum, jejunum, etc.), we did not detect any major differences or heterogeneity among the many SI samples examined. In addition, while our samples ranged in age from 20–23 weeks, this variable was deemed non-essential due to proper clustering of gene expression among the SI and LI samples.

We then used Ingenuity Pathway Analysis Software to predict expression changes in the dataset that relate to biological processes and disease. Functional differences in gene expression between SI and LI appeared frequently in known cancer and gastrointestinal disease pathways. The transcripts with the highest fold changes between SI and LI were related to proliferation, adhesion, migration and those that have reported functions in cancer and metastasis. Tumor-related transcripts were expressed significantly higher in cells expanded from the LI compared to SI (Fig. 3B). We also found differences in Wnt and Notch pathway transcripts (Fig. 3C&D). Real-time PCR of selected genes confirmed the differential expression found with the gene array analysis (S2 Fig.). Together, expression analysis of expanded cells indicated distinct intrinsic regulation between SI and LI at the stem cell level.

### Expanded cells from SI and LI demonstrated multipotentiality and responded differently to exogenous cues in three-dimensional culture

**Fig 4:**
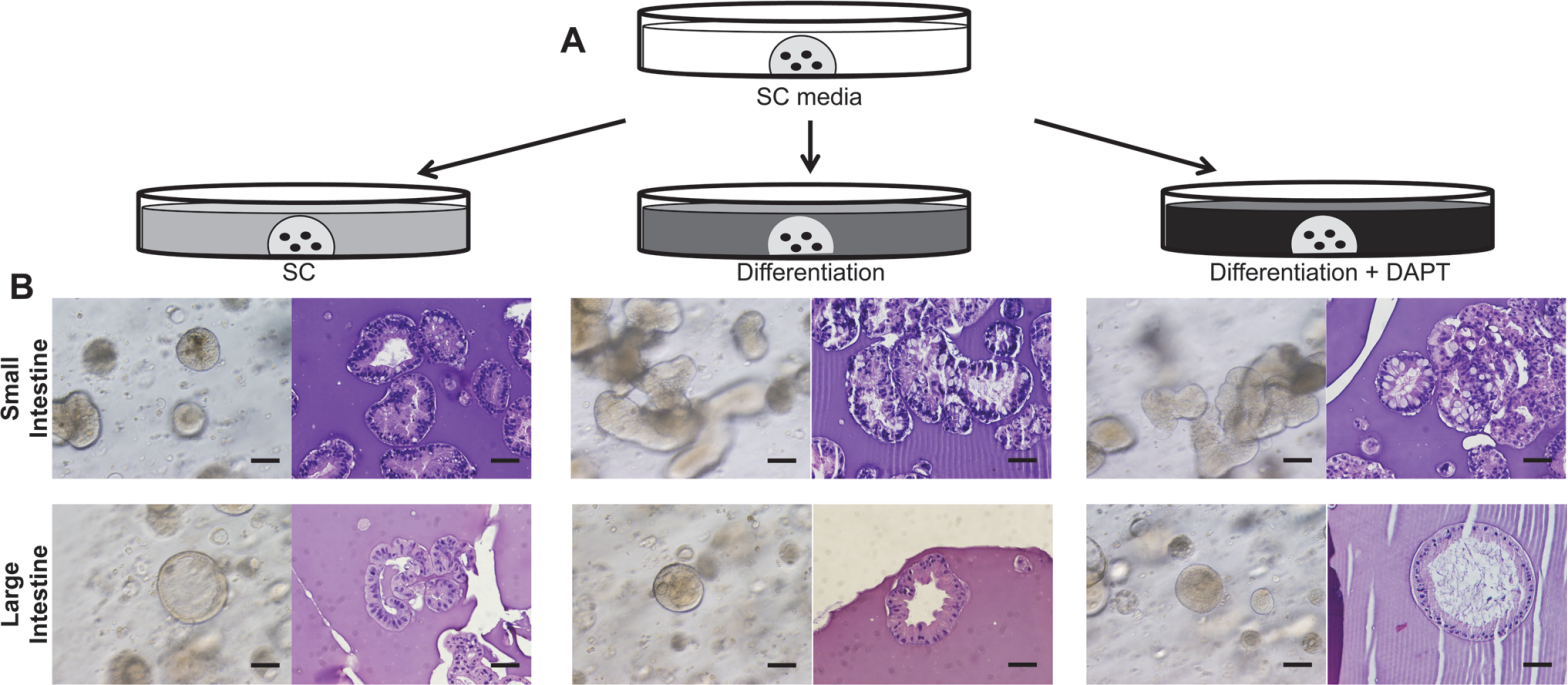
Three-dimensional differentiation assay for cells expanded from SI and LI. (**A**) Experimental design of three-dimensional assay. Expanded intestinal cells were embedded as single cells in Matrigel, first expanded in Stem Cell (SC) media, then treated further with SC media, Differentiation media or Differentiation media + DAPT (GSI). (**B**) *In vitro* and H&E pictures of SI (top) and LI (bottom) organoids in each of the three treatments. One paired set of SI and LI organoids is representative of three sets examined. Scale bar = 50μm.

Intestinal stem cells are partially defined by their multipotentiality, so we investigated the ability of expanded intestinal cells to differentiate into various intestinal cell lineages. We adapted a previously defined three-dimensional culture system to promote cell differentiation *in vitro* [5]. After expansion on the feeder layer to eliminate differentiated cells and create a more homogenous cell population, single cell suspensions were embedded in Matrigel and overlaid with media containing growth factors known to be important for the proliferation and differentiation of intestinal stem cells [19] (see Methods and Fig. 4A). Use of a γ-secretase inhibitor (GSI), such as N-[N-(3,5-difluorophenacetyl)-1-alanyl]-S-phenylglycine t-butyl ester (DAPT), blocks the translocation of the Notch Intracellular Domain (NICD) to the nucleus for downstream signaling, causing forced differentiation to goblet cells [16,19,31]. Expanded intestinal cells retained the ability to differentiate and form a layer of epithelial cells around a central lumen with a cellular hierarchy and architecture similar to that found *in vivo* (Fig. 4B). Careful tracking of single cells showed growth into multicellular organoids, which supports the clonal origin for at least some of the SI and LI organoids (S3 Fig.). Furthermore, we observed morphological differences between SI and LI organoids with the appearance of branching structures in the SI and spherical structures in the LI (Fig. 4B).

**Fig 5:**
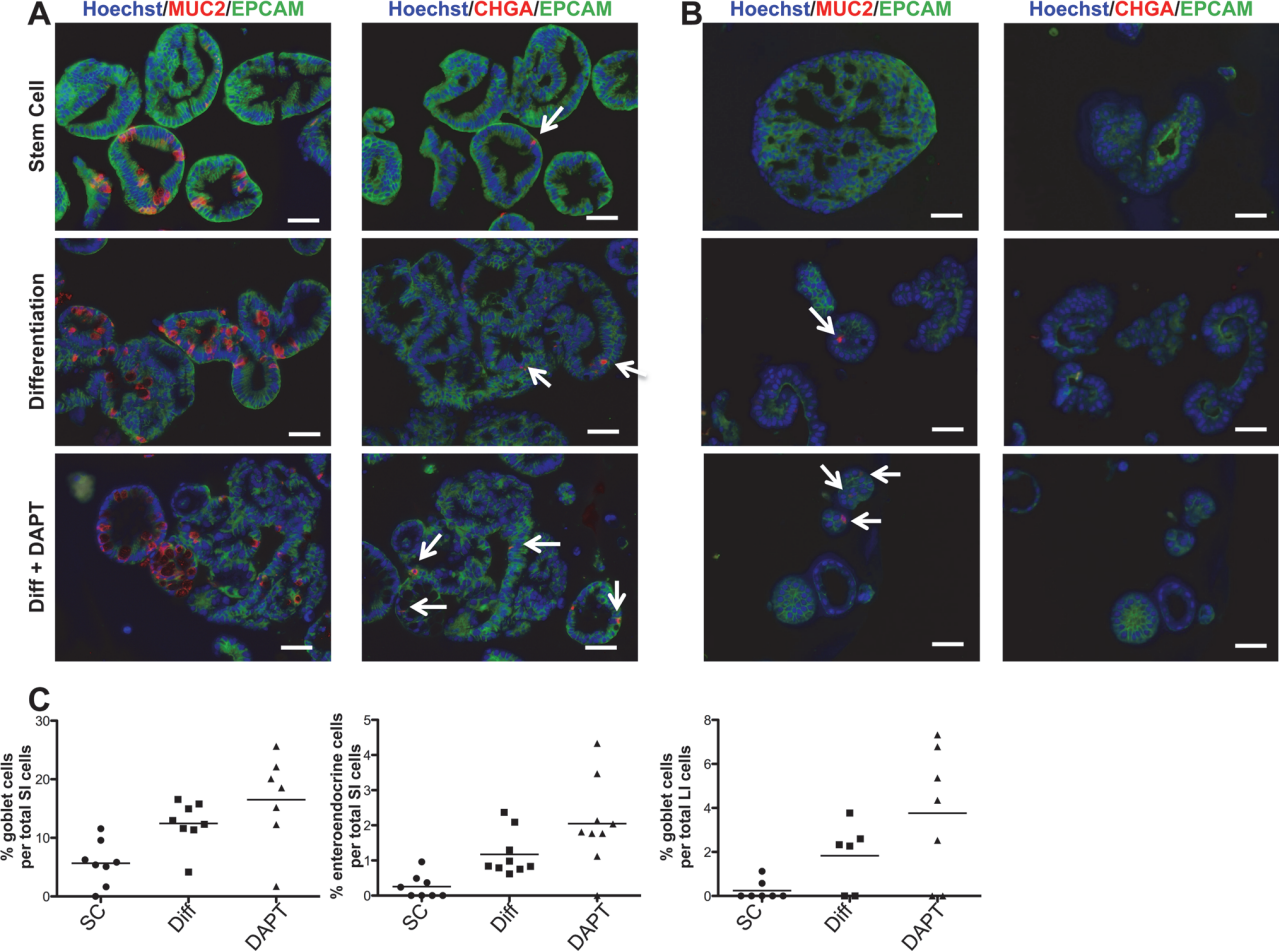
Immunofluorescent staining of intestinal organoids confirms multipotentiality and differentiation potential. (**A**) SI and (**B**) LI organoids from one paired sample stained for epithelial marker, EPCAM (green), goblet cell marker, MUC2 (red) and enteroendocrine marker, CHGA (red) treated with SC media, Differentiation media (Diff) or Diff media + DAPT. Counterstain, Hoechst 33342. White arrows indicate positive cells. Scale bar = 50μm. (**C**) Quantification of percent goblet or enteroendocrine cells of total cells counted per field of view. Not enough enteroendocrine cells were present in the organoids derived from LI for quantification. This paired set serves as a representative of two sets examined.

Using immunofluorescent staining for differentiation markers, we analyzed SI and LI organoids for the presence of mature intestinal cells. The expression of Mucin 2 (MUC2), a marker of mature goblet cells, significantly increased upon treatment with differentiation media without and with DAPT (Diff and Diff+DAPT) in both SI and LI organoids (Fig. 5A-C). This correlated with the appearance of vacuolated cells in both differentiation treatments in SI organoids (Fig. 4B). SI organoids also showed increased expression of Chromogranin A (CHGA), a marker for enteroendocrine cells, upon differentiation treatment (Fig. 5A&C). Under the same conditions, LI organoids failed to express any measurable amounts of CHGA (Fig. 5B). A similar phenomenon was also seen *in vivo* where a significantly higher number of CHGA^+^ cells was observed in SI compared to LI (S4 Fig.). Furthermore, enterocyte expression of Villin (VIL1) in SI organoids was expressed mostly in the cytoplasm but was specifically localized on the apical cell surface in differentiated LI organoids (S5 Fig.).

### Gene expression in SI and LI organoids differed in response to differentiation stimuli

**Fig 6:**
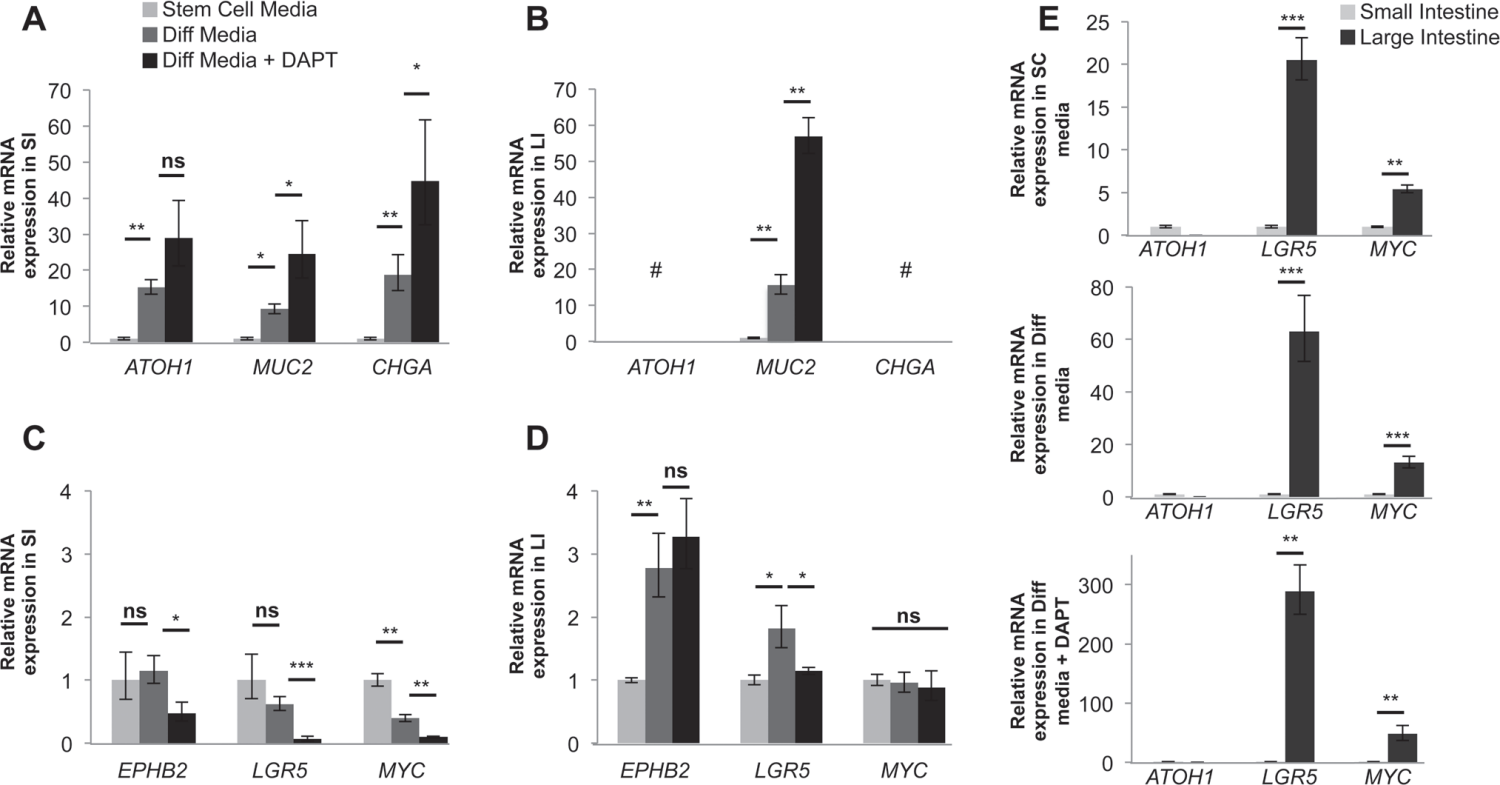
Small and large intestine organoids differ in response to differentiation cues. Real-time PCR analysis of (**A&C**) SI and (**B&D**) LI organoids derived from expanded cells and treated with SC media, Differentiation (Diff) or Diff media + DAPT. SC media treatment values used as control and set to 1. Diff media and Diff media + DAPT expression values are relative to SC media. See media key in panel A. (**E**) Select marker expression comparison between SI and LI organoids in the three treatments. SI values used as control and set to 1. LI organoid expression values are relative to SI organoids. See text for full gene names. Expression was normalized to *GAPDH* mRNA. Error bars represent upper and lower error limits based on replicate variability (*P<0.05, **P<0.01, ***P<0.001, # no expression detected). (n = 3 wells per sample/primer pair). One paired SI and LI set serves as a representative of all paired sets examined.

Real-time PCR showed increased relative mRNA expression of *ATOH1* and *MUC2* in SI organoids upon differentiation treatment (Fig. 6A). In LI organoids, *MUC2* expression increased upon exposure to differentiation cues, while *ATOH1* expression was negligible (Fig. 6B). Additionally, while *CHGA* expression steadily increased with differentiation treatment in SI organoids, no appreciable *CHGA* expression was observed in LI organoids, confirming our immunofluorescence data (Fig. 6B and Fig. 5A&B). Furthermore, as expected, the expression of stem cell marker and Wnt pathway participant *LGR5* decreased in SI organoids upon differentiation treatment. Surprisingly, however, *LGR5* reproducibly increased in LI organoids after the addition of Diff media, and only returned to stem cell (SC) media levels after treatment with DAPT (Fig. 6C&D). We also found that EPH receptor B2 (*EPHB2*), another putative intestinal stem cell marker [32], consistently increased upon Diff treatment of LI organoids. In SI organoids, *EPHB2* remained unchanged in Diff media but subsequently decreased with the addition of DAPT. In addition, the *MYC* oncogene, a Wnt target, also decreased after exposing SI organoids to differentiation treatment, but a significant change of expression in LI organoids was not observed.

We then compared SI and LI organoids directly at each of the three treatment steps and again found undetectable expression of *ATOH1* in LI organoids while SI organoids displayed substantial expression of *ATOH1*. *LGR5* and *MYC* expression were significantly higher in LI organoids compared to SI organoids in all matrigel conditions (Fig. 6E). Together, the results from the differentiation assay support the gene array analysis (Fig. 3C&D) and demonstrate that the cells expanded from SI and LI have the stem cell property of multipotentiality but differ in their response to the same environmental cues.

## Discussion

In this study, we used paired sets of human fetal SI and LI to investigate the differences between the two distinct anatomic regions with potentially different stem cell populations. While others have successfully cultured organoids from mouse and/or human tissue from varying ages [5,19,27,32], excluding 20–23 week human fetal tissue as used here, none directly compared the intrinsic differences of the SI and LI. *In vivo*, a combination of intrinsic and extrinsic factors determine the structural and cellular components of the SI and LI [33]. Here, we have removed any differences present in the SI and LI environments by providing identical growth factors and culture conditions for SI and LI cells. Therefore, the differences seen in cell lineage specification *in vitro* are due to differential intrinsic regulation of stem cells present in SI and LI.

We limited our investigation to human cells and by culturing bulk intestinal cells on a feeder layer with exogenous Wnt factor, R-Spondin 2, we were able to characterize expanded epithelial cells for stem cell properties such as self-renewal and mulitpotentiality without bias of pre-selecting the cells for a particular phenotype. *In vivo* balance of proliferation and differentiation of the intestinal epithelium, including the stem cell niche, depends on many Wnt factors [4,5,34]. Only recently was high expression of R-Spondin 2, known to enhance intestinal epithelial precursors and limit differentiation via LGR5, found in intestinal subepithelial myofibroblasts [35,36]. Therefore, the R-Spondin 2 dependence of the intestinal epithelial cells expanded on feeder cells *in vitro* is consistent with the maintenance of intestinal stem cells *in vivo*.

The expected shared expression of putative stem cell markers CD133.1, CD166 and CD24 on cells expanded from the two intestinal segments suggested that they were of stem cell nature. However, differences emerged after analysis of the specific expression patterns of CD13, CD133.1 and CD66c. A higher expression of CD13 in cells expanded from SI is consistent with reports showing that it is the most abundant peptidase in mammalian SI, with CD13 expression continually decreasing along the ascending and descending LI [37,38]. Furthermore, considering its relation to colon CSCs, the higher expression of CD133.1 on cells expanded from LI may indicate a predisposition of these cells to neoplastic transformation compared to cells expanded from SI [39]. Finally, by functionally exploiting the differential expression pattern of CD66c, we identified opposing populations of cells with colony-forming ability. High CD66c expression in stem cell enriched cultures from the LI confers increased self-renewal and, together with high expression of CD133.1, suggests an intrinsic regulatory difference for why these stem cells may be more susceptible to transformation than their SI counterparts [40,41].

Furthermore, gene array analysis demonstrated global differences between cells expanded from the SI and LI. Differential expression indicated a tendency for cells expanded from the LI to be more closely related to cancer than their SI counterparts. Specifically, serologically defined colon cancer antigen 1 (*SDCCAG1*) and tumor-associated calcium signal transducer 2 (*TACSTD2*) are defined by their presence and association with cancer. Hypoxia inducible factor 1 (*HIF1A*) functions in angiogenesis, ready to feed any new tumor that begins to form [42]. *Prom1*, also known as *CD133*, is a well-known CSC marker discussed previously [20]. The higher expression of insulin-like growth factor binding protein 3 (*IGFBP3*), involved in intestinal epithelium homeostasis, may indicate a more delicate balance necessary in the LI compared to the SI [43]. Lastly, secreted phosphoprotein 1 (*SPP1*), also known as osteopontin (*OPN*), was the transcript with the highest fold change between LI and SI cells (142-fold higher in LI cells). Not only is SPP1 expression higher in colorectal cancer cell lines than normal intestinal cell lines, but it is higher in tumor tissue in comparison to normal tissue [44,45]. It also correlates with lymph node metastasis, lymphatic or venous invasion and TNM stage (classification of malignant tumors). Patients with a higher expression of SPP1 had lower 5-year survival rates and it is thought to be an independent prognostic factor. Therefore, it may play a role in CRC progression and could be a prognostic marker for patients [45]. The increased expression of these molecules in LI cells and their associated molecular pathways not only confirm the intrinsic differences between the SI and LI, but possibly a functional reason for why there is more cancer in the LI compared to the SI. Therefore, differences at the stem cell level not only contribute to the separate structure and function of the two tissue segments, but also to their differing response to insults leading to disease.

The molecular differences identified between cells expanded from the SI and LI correlated well with the disparities in differentiation using a three-dimensional culture system. Fordham et. al used similar culture conditions for the growth of intestinal stem cells from fetal material at the gestational age of ten weeks [46]. However, cells from such an early stage did not require expansion prior to high efficiency formation of organoids from primary tissue, but did require additional factors to sustain cells. At 20–23 weeks, as presented here, the primary tissue structure is much more differentiated, mimicking adult organization and requiring selection and expansion on feeder cells prior to use in organoid formation assays. In addition, in the Fordham et. al study, whole epithelial units were initially embedded in their cultures, whereas in our study, single cells were plated on a feeder layer in order to eliminate the more differentiated cells present at 20–23 weeks gestational age. The feeder layer allowed us to expand and maintain the stem cell population in an undifferentiated state prior to embedding in Matrigel. This expansion allowed for a more homogenous cell population for analysis in the organoid system, without the presence of differentiated cells from primary tissue.

The initial observation of differentiation into goblet cells confirmed the multipotentiality of cells expanded from both SI and LI while demonstrating that their intrinsic differences determine unique differentiation potential. The undetectable expression of *ATOH1* in LI organoids was in agreement with the gene expression profiling data, which showed that cells expanded from LI express *ATOH1* at a lower level than cells expanded from SI. Interestingly, *ATOH1* is required for the efficacy of GSIs [47], which may explain the limited MUC2 protein expression seen in DAPT treated LI organoids. Previous studies showed that 70% of colorectal cancer patient samples have significantly decreased expression of ATOH1 compared to tissue-matched normal LI samples, and revealed ATOH1 as a tumor suppressor *in vitro* and *in vivo* [48]. Therefore, the lack of detectable *ATOH1* in cells expanded from the LI could make them more susceptible to transformation than their SI counterparts.

Lastly, the disparity in expression of stem cell related genes *EPHB2*, *LGR5* and *MYC* might indicate a yet unknown molecular pathway that distinguishes LI stem cells from SI stem cells. The higher expression of *LGR5* in LI organoids could mean the presence of cells with higher resistance to damage, DNA repair and growth advantage that, if transformed, could confer resistance to cancer cells [49,50]. Even though *LGR5* expression remains high with differentiation treatment in LI cultures, LGR5 likely remains a stem cell marker in both the SI and LI. *Lgr5* ^+^ cells are present in cycling columnar cells of the mouse at the crypt base and can generate cells of all the epithelial lineages of the intestine, suggesting stem cell nature [4]. In addition, as a receptor for R-Spondin 2, LGR5 can mediate the stem cell niche [6,8]. However, the work presented here presents a novel observation in human cells of differential regulation of *LGR5* upon differentiation that may indicate a separate function of LGR5 in the LI in addition to its presence as an intestinal stem cell marker.

Functionally, LGR5 is both a receptor for Wnt ligands (R-spondins) and a transcriptional target of the Wnt pathway [4,6,8]. Here, we show that *LGR5* mRNA is expressed differently between SI and LI cells, with *LGR5* remaining high in LI cells after the removal of Wnt factors and even under γ-secretase inhibition (Fig. 6D&E). This seems to be discordant with a parallel increase in goblet cells under the same conditions in LI cells after differentiation treatment (Fig. 5A-C). Wnt and Notch signaling pathways are known to function cooperatively in intestinal epithelium, intestinal stem cells and tumor development [1,13,14]. ATOH1, a key regulator of the Notch pathway, can be marked for degradation by GSK3-β in the presence of Wnt signals and is thought to be necessary for goblet cell formation and response to γ-secretase inhibition [47]. Here, we show that *ATOH1* was not detected in cells derived from LI, even under γ-secretase inhibition (Fig. 6B&E), suggesting an alternative pathway in the LI. The combined result of high *LGR5* coupled with the absence of *ATOH1* in the LI, may indicate a possible new pathway potentially involving LGR5/Wnt and ATOH1/Notch for the regulation of differentiation in the LI.

To our knowledge, this is the first comprehensive report characterizing the differences between cells expanded from primary human SI and LI. The expected results observed from experiments in SI samples confirm that combining the expansion of an enriched stem cell population with subsequent three-dimensional differentiation assays reproducibly creates an environment similar to that found *in vivo*. While there is evidence of extrinsic regulation of intestinal stem cells [33] the unexpected results seen with the LI samples suggest key intrinsic differences between human SI and LI stem cells. These differences may account for the increased prevalence of LI cancer compared to SI cancer, as well as highlight crucial protective mechanisms present in the SI that could be harnessed to improve prevention and treatment of human LI cancer.

## Methods

### Ethics Statement

Mouse experiments were performed under the guidelines of the University of Pittsburgh Institutional Animal Care and Use Committee, which approved the study, and were performed in accordance with the NIH guidelines for the care and use of animals (approval no. 1006567). Human fetal intestines were obtained through Magee Women’s Hospital and the University of Pittsburgh Health Sciences Tissue Bank with approval from the University of Pittsburgh Institutional Review Board (IRB approval no. PRO10090385) after written consent.

### Preparation of feeder cells

LA7 (ATCC: CRL-2283) cells were grown in DMEM/F12 (Mediatech, VA) supplemented with 5% FBS, 1% Pen/Strep (Mediatech, VA), 50nM hydrocortisone (Sigma-Aldrich, MO) and 5μg/mL Insulin (Sigma-Aldrich, MO). Cells were detached from the plate with 0.25% Trypsin/0.1% Ethylenediaminetetraacetic acid (EDTA) (Mediatech, VA) and γ-irradiated at 17,000 rads. Cell culture flasks were seeded at ∼300,000 cells/cm^2^ to generate a confluent monolayer of feeder cells prior to seeding human cells.

### Intestinal cell isolation and culture

Small and large intestines were obtained from 19–23 week human fetuses. Intestines were cut longitudinally in HBSS, contents rinsed, cut into 1-inch pieces, transferred to EBSS/1mM EGTA/1% HEPES (Life Technologies, NY/Sigma-Aldrich, MO/Mediatech, VA) and minced. Tissue was then incubated for 5 min at room temperature. After an EBSS wash, the tissue was treated three times with a cocktail containing 1mg/mL collagenase II (Life Technologies, NY), 1mg/mL hyaluronidase (Sigma-Aldrich, MO) and 20μg/mL DNase I (Roche, IN) in HBSS/1% HEPES for 20 min. Tissue/cell suspensions were passed through a 100μm cell strainer (Fisher, PA) to isolate single cells from undigested tissue. Cells were plated on irradiated feeders at ∼80,000 cells/cm^2^ in DMEM/F12 supplemented with 0.5% FBS, 25μg/mL gentamicin (Sigma-Aldrich, MO), 1% Insulin-Transferrin Selenium (ITS) (Mediatech, VA) and 0.1ng/mL human R-Spondin2 (R&D, MN). When single cells were plated, 10μM ROCK inhibitor, Y-27632 (Reagents Direct, CA), was added to the media for ∼24 hours. Cultures were passaged at 2–3 weeks post-plating (∼70% confluence) by incubating with EBSS/1mM EGTA/1% HEPES followed by 0.25% Trypsin/0.1% EDTA.

### Fluorescence-Activated Cell Sorting and Analysis

Single cell suspensions were stained with appropriate antibodies (S3 Table) at 200,000 cells per tube and analyzed on the MACSQuant (Miltenyi Biotec, Germany) or BD FACSAriaII (BD Biosciences, MA). Singlet discrimination was performed as described by Wersto et al. [51]. Post-acquisition analysis was carried out in FlowJo (http://www.treestar.com). Staining index was calculated as D/W, where D is the distance between the positive and negative populations and W is two standard deviations of the negative population. LDAs were performed by sorting 1, 10, 25, 50, 100, 250, 500, 1000 cells per well into respective rows of 96-well plates (Corning, NY) previously seeded with irradiated feeder cells. Individual wells were scored for the presence of colonies at four weeks. The number of wells with colonies was input into L-Calc (Stem Cell Technologies, Vancouver, BC, Canada) to calculate CFU frequency.

### Matrigel Differentiation Assay

The differentiation assay used in this study was modified from Sato, et al. [19]. Briefly, expanded intestinal cells were suspended in 50μL Growth Factor Reduced (GFR), phenol-red free Matrigel (BD Bioscience, CA) and plated in tissue culture dishes at low density (∼50,000 cells/well of 48-well plate). After solidification at 37°C for 30 min, the Matrigel was overlaid with Stem Cell media (SC) (S4 Table). For the first 24 hours, the media included Y-27632 to prevent anoikis. After initial plating in SC media and the formation of small organoids, two-thirds of the wells were switched to Differentiation (Diff) media to allow differentiation of the organoids to occur. After further growth of the organoids, DAPT (Reagents Direct, CA), a γ-secretase inhibitor, was added to half the wells containing Diff media at 10μmol/mL for three days. Media was changed on all wells three times a week. Resulting organoids were isolated using 0.2% dispase (Life Technologies, NY), 0.1% collagenase II and 20μg/mL DNase I. Organoids were then rinsed in PBS (Mediatech, VA) for downstream applications.

### mRNA Expression Assays

Total RNA was extracted from frozen cell pellets of expanded human fetal cells from SI or LI (SI; n = 6 and LI; n = 6) ranging from 2–8 passages on the feeder cells. Expression data (q value<0.05, 2 fold change) was imported into Ingenuity software (Ingenuity Pathways Analysis, Ingenuity Systems Inc, CA) for pathway analysis. All figures are plotted using unlogged data to maintain the original biological distribution of expression. See S1 Methods for more detail.

## Supporting Information

S1 Fig Depiction of the Notch pathway. Notch ligand, Delta/Serrate/LAG-2 (DSL), of one cell activates a Notch receptor on a neighboring cell. Notch intracellular domain (NICD) is cleaved by gamma secretase (GS) and activates Hes1. Hes1 blocks Muc2 activation via blockage of ATOH1 and the cell retains a progenitor state. In the cell where Notch is inactive, ATOH1 activates MUC2 and the cell adopts a goblet cell fate.
(TIF)

S2 Fig Real-time PCR confirmation of gene array analysis. Selected genes were assayed using real-time PCR as confirmation of gene array results. RNA from cells expanded from three paired SI and LI samples (1, 2 and 3) were assayed for expression of six genes identified as differentially expressed in the gene array analysis. In 6/6 cases, the real-time PCR analysis confirmed the same expression pattern as the gene array analysis. SI values used as control and set to 1 in each case. LI expression values are relative to SI expression. SI 1 and 3 had no detectable expression of *SPP1*, therefore, SI2 was used to calculate fold change in LI 1, 2 and 3. Expression was normalized to *GAPDH* mRNA. Fold changes are presented above bars for clarification. Error bars represent upper and lower error limits based on replicate variability. All expression comparisons between SI and LI cells were significant (*P*<0.05). (n = 3 wells per sample/primer pair).
(TIF)

S3 Fig Tracking organoid growth from a single cell. SI or LI organoids grown in SC media, Differentiation (Diff) media and Diff media supplemented with DAPT for the last three days, followed from a single cell to multicellular organoids. Arrows and arrowheads indicate the growth of organoids from a single cell. Scale bar = 50μm.
(TIF)

S4 Fig Enteroendocrine cells are found more frequently in the SI compared to the LI *in vivo*. (**A**) Normal human fetal SI (left) and LI (right) stained for epithelial marker, EPCAM (green), and enteroendocrine marker, CHGA (red). Counterstain, Hoechst 33342. Scale bar = 50μm. (**B**) Cells were counted as cells per crypt and depicted in a bar graph (*P<0.05).
(TIF)

S5 Fig Differential expression of Villin in intestinal organoids. SI (left) and LI (right) organoids grown in SC media, Differentiation (Diff) media and Diff media supplemented with DAPT stained for enterocyte marker, Villin (VIL1) (red). Counterstain, Hoechst 33342. Scale bar = 50μm.
(TIF)

S1 Table Expanded normal intestinal cells cultured with R-spondin 2 do not form tumors *in vivo*. (PDF)

S2 Table Gene names of symbols used in Fig. 3. (PDF)

S3 Table Comprehensive list of antibodies used for flow cytometry. (PDF)

S4 Table Comprehensive list of reagents used for organoid cultures. (PDF)

S5 Table Comprehensive list of antibodies used for immunofluorescence. (PDF)

S6 Table Comprehensive list of primers used for Real-Time PCR. (PDF)

S1 Methods A more comprehensive description of methods used. (DOCX)
