# Supplementary figures and images for: Distinct Human Stem Cell Populations in Small and Large Intestine
Source: PLoS One. 2015 Mar 9;10(3):e0118792. doi: 10.1371/journal.pone.0118792 (PMC4353627; doi:10.1371/journal.pone.0118792)

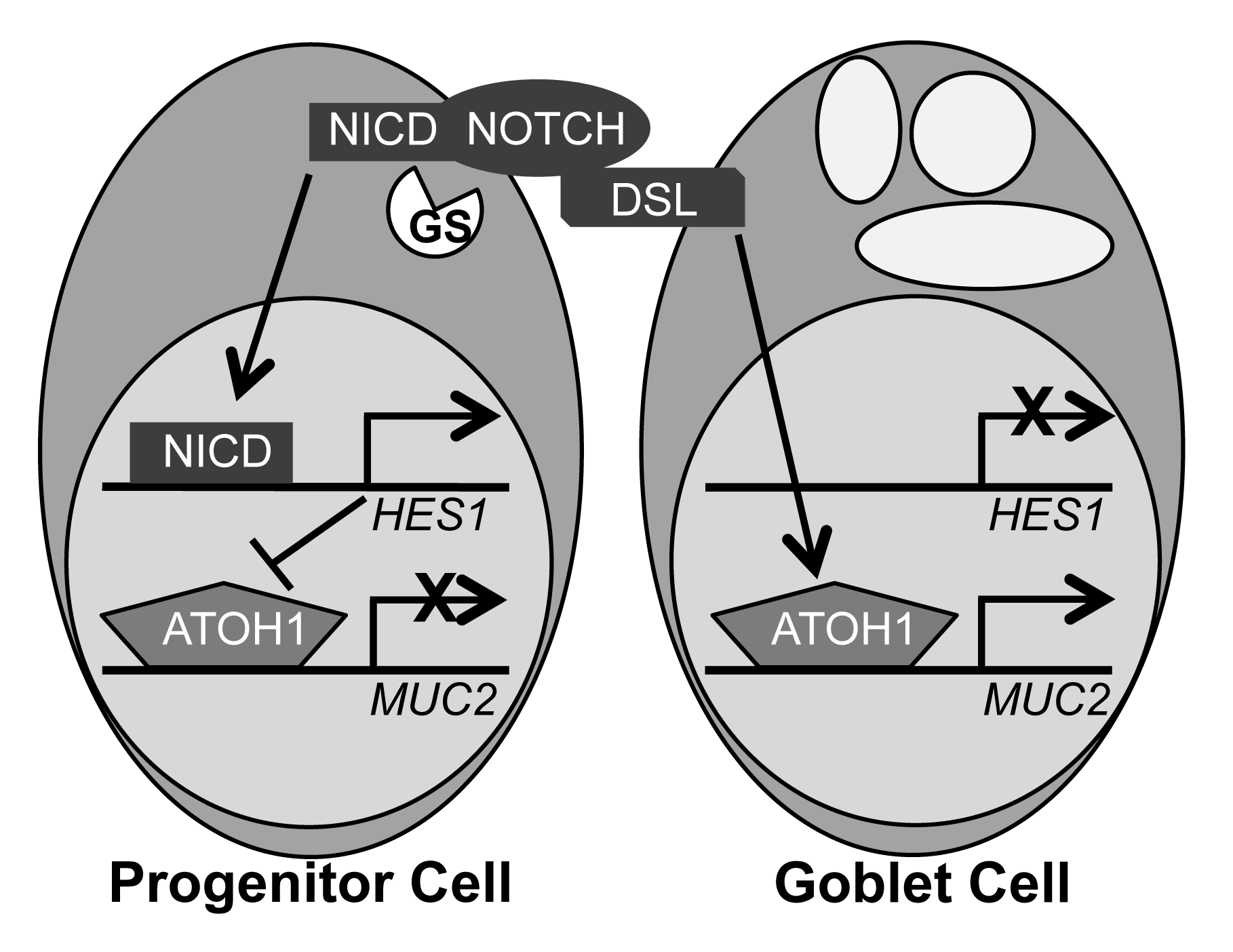

Supplement: S1 Fig — Notch ligand, Delta/Serrate/LAG-2 (DSL), of one cell activates a Notch receptor on a neighboring cell. Notch intracellular domain (NICD) is cleaved by gamma secretase (GS) and activates Hes1. Hes1 blocks Muc2 activation via blockage of ATOH1 and the cell retains a progenitor state. In the cell where Notch is inactive, ATOH1 activates MUC2 and the cell adopts a goblet cell fate. (TIF) [file pone.0118792.s001.tif]

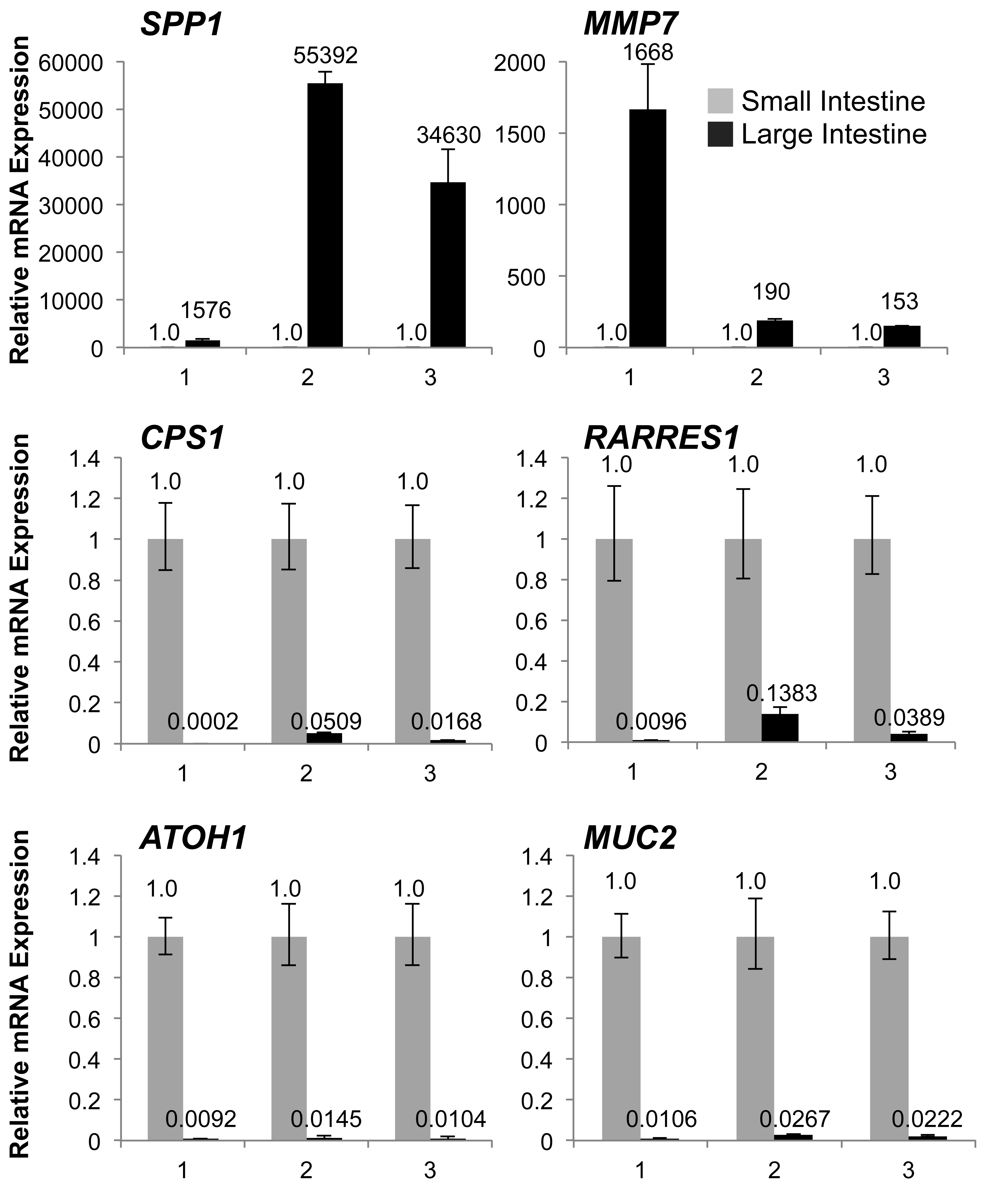

Supplement: S2 Fig — Selected genes were assayed using real-time PCR as confirmation of gene array results. RNA from cells expanded from three paired SI and LI samples (1, 2 and 3) were assayed for expression of six genes identified as differentially expressed in the gene array analysis. In 6/6 cases, the real-time PCR analysis confirmed the same expression pattern as the gene array analysis. SI values used as control and set to 1 in each case. LI expression values are relative to SI expression. SI 1 and 3 had no detectable expression of SPP1, therefore, SI2 was used to calculate fold change in LI 1, 2 and 3. Expression was normalized to GAPDH mRNA. Fold changes are presented above bars for clarification. Error bars represent upper and lower error limits based on replicate variability. All expression comparisons between SI and LI cells were significant (P<0.05). (n = 3 wells per sample/primer pair). (TIF) [file pone.0118792.s002.tif]

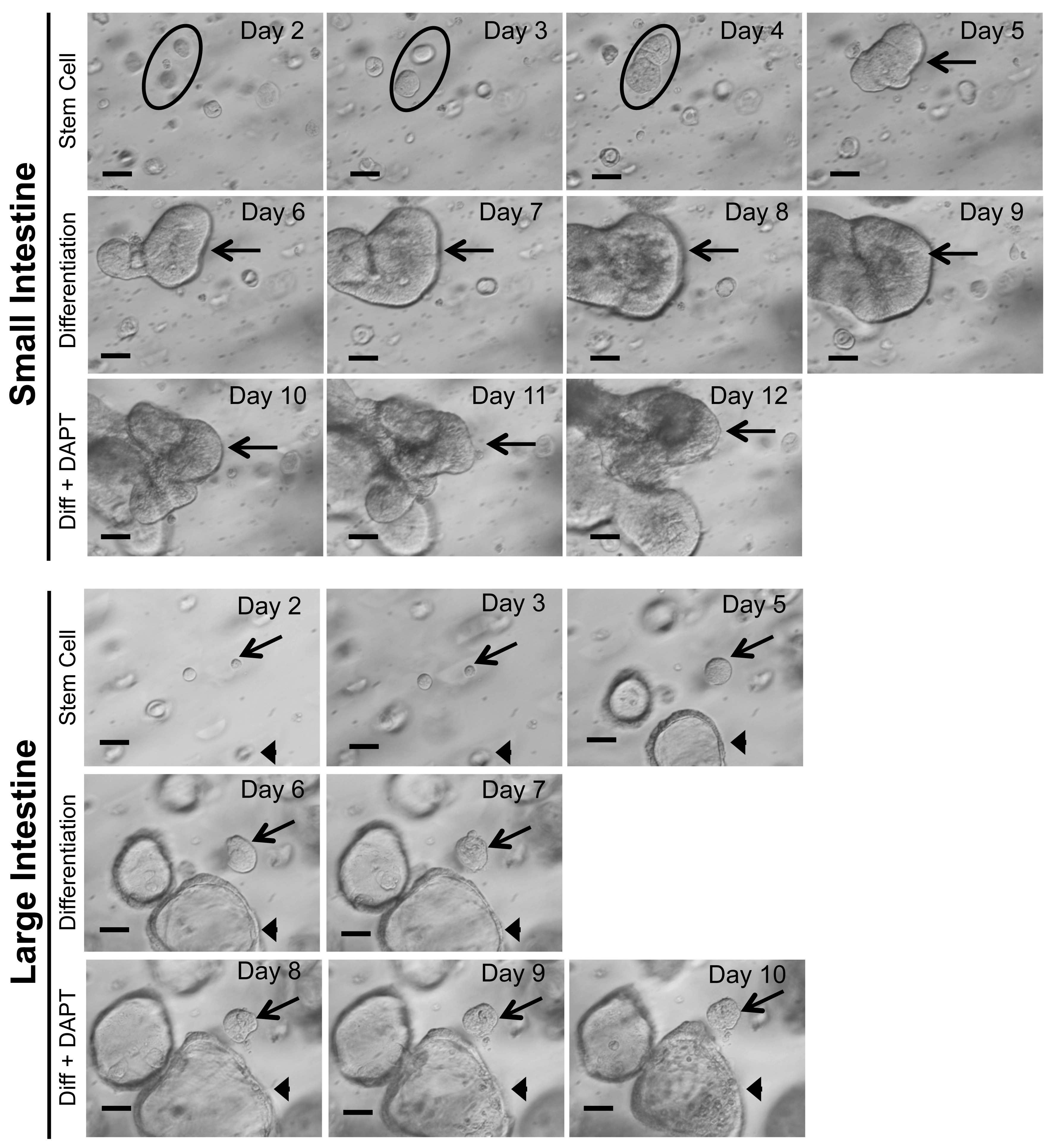

Supplement: S3 Fig — SI or LI organoids grown in SC media, Differentiation (Diff) media and Diff media supplemented with DAPT for the last three days, followed from a single cell to multicellular organoids. Arrows and arrowheads indicate the growth of organoids from a single cell. Scale bar = 50μm. (TIF) [file pone.0118792.s003.tif]

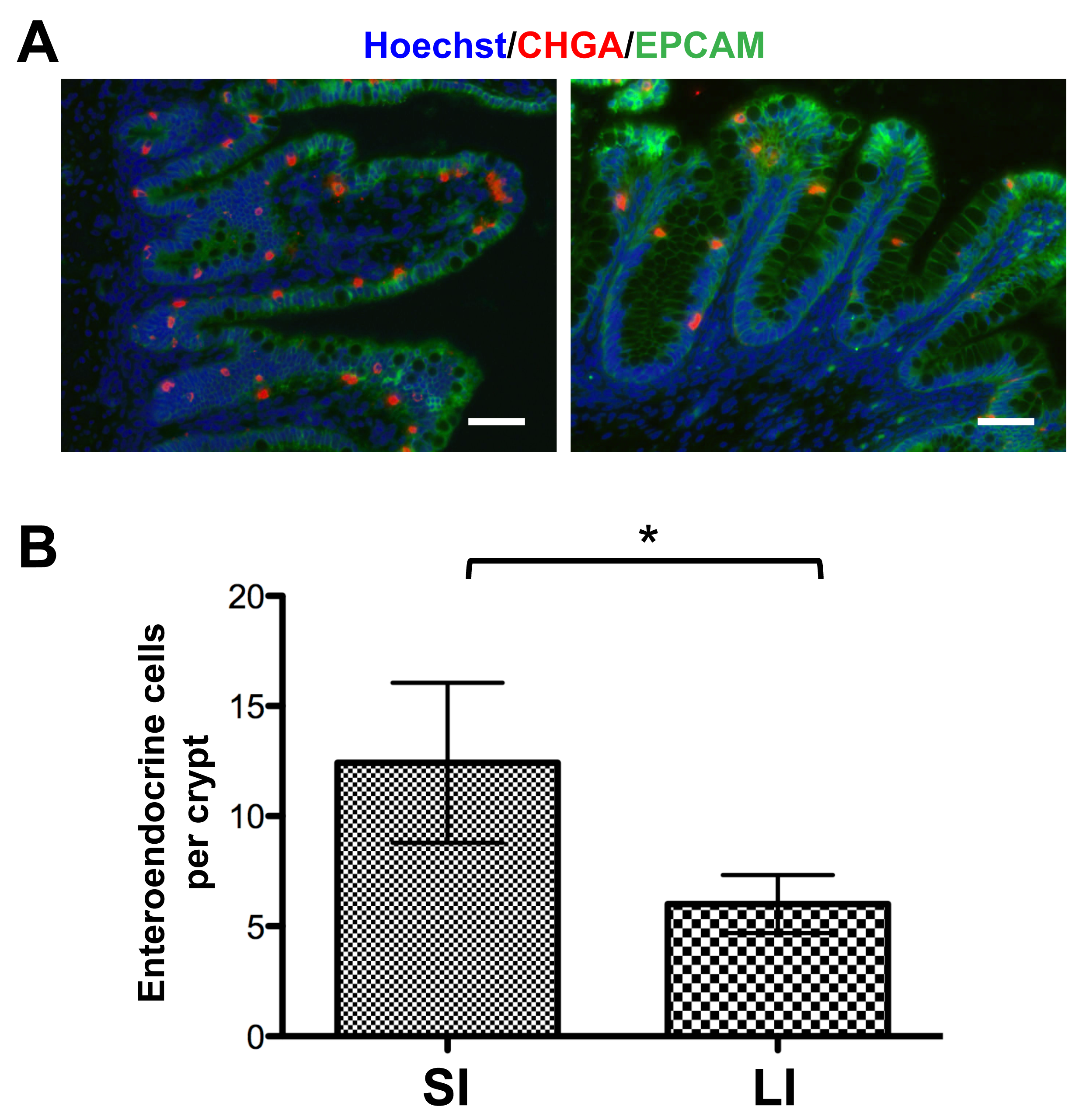

Supplement: S4 Fig — (A) Normal human fetal SI (left) and LI (right) stained for epithelial marker, EPCAM (green), and enteroendocrine marker, CHGA (red). Counterstain, Hoechst 33342. Scale bar = 50μm. (B) Cells were counted as cells per crypt and depicted in a bar graph (*P<0.05). (TIF) [file pone.0118792.s004.tif]

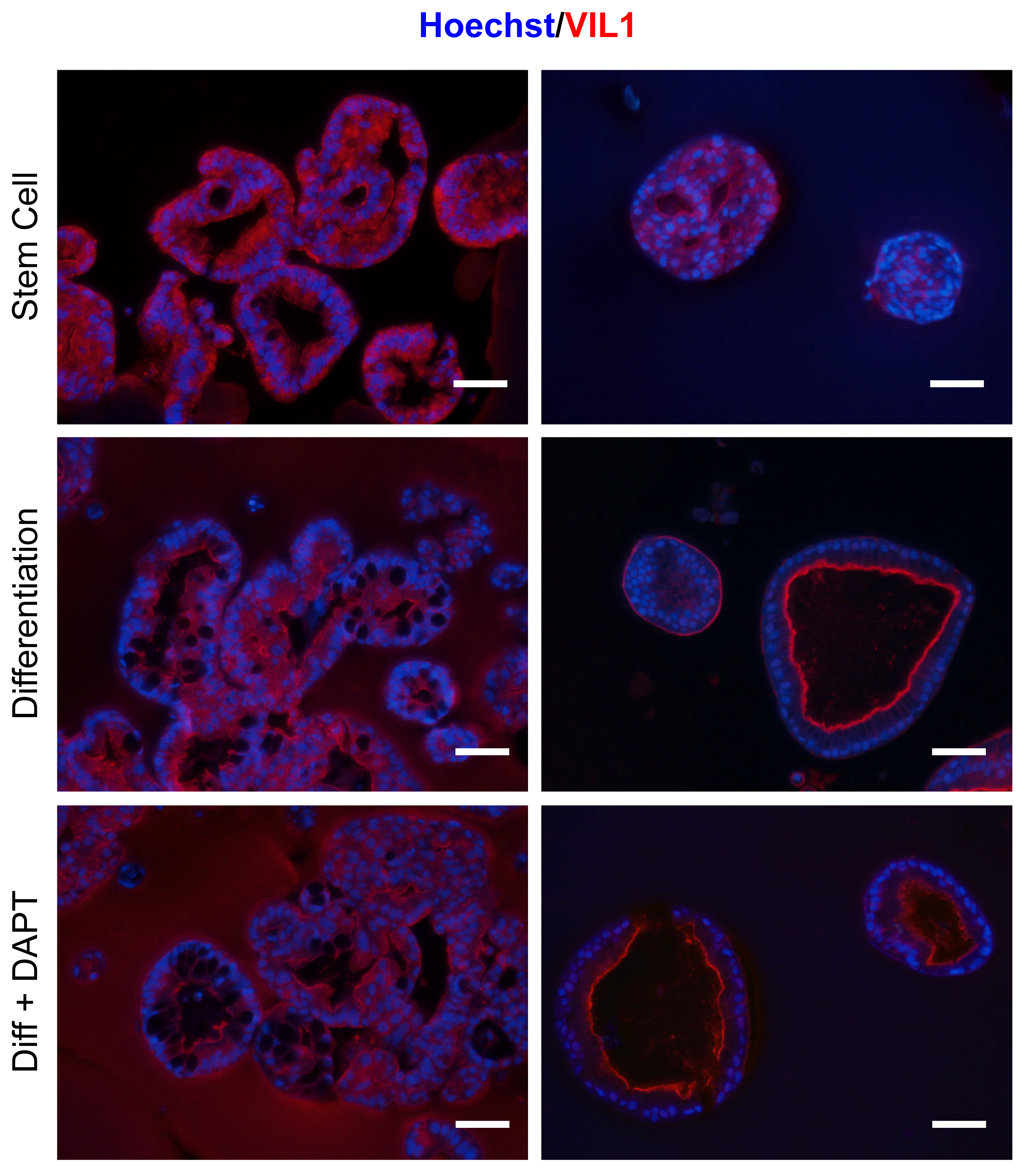

Supplement: S5 Fig — SI (left) and LI (right) organoids grown in SC media, Differentiation (Diff) media and Diff media supplemented with DAPT stained for enterocyte marker, Villin (VIL1) (red). Counterstain, Hoechst 33342. Scale bar = 50μm. (TIF) [file pone.0118792.s005.tif]
