# Supplementary material for: Distinct Human Stem Cell Populations in Small and Large Intestine
Source: PLoS One. 2015 Mar 9;10(3):e0118792. doi: 10.1371/journal.pone.0118792 (PMC4353627; doi:10.1371/journal.pone.0118792)
Supplement: S1 Table — (PDF) [file pone.0118792.s006.pdf]

S1_Table

Cell Type	Frequency of Tumor Formation
SI cells	0/5
LI cells	0/6
Colon CSCs	5/5
