# Supplementary material for: Distinct Human Stem Cell Populations in Small and Large Intestine
Source: PLoS One. 2015 Mar 9;10(3):e0118792. doi: 10.1371/journal.pone.0118792 (PMC4353627; doi:10.1371/journal.pone.0118792)
Supplement: S2 Table — (PDF) [file pone.0118792.s007.pdf]

S2 Table

Symbol	Gene name
ATOH1	atonal homolog 1
CCND1	cyclin D1
CD44	CD44 molecule (Indian blood group)
CHGA	chromogranin A
FZD6	frizzled homolog 6
IGFBP3	insulin-like growth factor binding protein 3
HIF1A	hypoxia inducible factor 1, alpha subunit
MMP7	matrix metallopeptidase 7
MUC2	Mucin2
NOTCH2	notch 2
PROM1	prominin 1
SDCCAG1	serologically defined colon cancer antigen 1
SOX4	SRY (sex determining region Y)-box 4
SPP1	secreted phosphoprotein 1
TACSTD2	tumor-associated calcium signal transducer 2
TLE1	transducin-like enhancer of split 1 (E(sp1) homolog
