# Supplementary material for: Distinct Human Stem Cell Populations in Small and Large Intestine
Source: PLoS One. 2015 Mar 9;10(3):e0118792. doi: 10.1371/journal.pone.0118792 (PMC4353627; doi:10.1371/journal.pone.0118792)
Supplement: S3 Table — (PDF) [file pone.0118792.s008.pdf]

S3 Table

Antibody	Antibody-conjugated fluorophore	Company	Clone	Concentration
CD24	FITC	BD Biosciences	ML5	1:50
CD66c	PE	BD Biosciences	B6.2/CD66c	1:50
CD13	PE	BD Biosciences	WM15	1:25
CD133.1	PE	Milteyni Biotec	AC133	1:50
CD166	PE	BD Biosciences	3A6	1:10
EPCAM	PerCP-Cy5.5	iCyt	9C4	1:50
HLA-Biotin	n/a	Ancell	3F10	1:100
Sav	APC-Cy7	BD Biosciences	Streptavidin	1:25
