# Supplementary material for: Distinct Human Stem Cell Populations in Small and Large Intestine
Source: PLoS One. 2015 Mar 9;10(3):e0118792. doi: 10.1371/journal.pone.0118792 (PMC4353627; doi:10.1371/journal.pone.0118792)
Supplement: S4 Table — (PDF) [file pone.0118792.s009.pdf]

S4 Table

Matrigel media components:	Final Concentration	Company	SC media	Diff Media
Advanced DMEM/F12	1x	Life Technologies	X	X
Pen/Strep	100/100 U/mL	Mediatech	X	X
HEPES	10mM	Mediatech	X	X
N2	1x	Life Technologies	X	X
B27	1x	Life Technologies	X	X
Glutamax	2mM	Life Technologies	X	X
Nacetyl-L-Cysteine	1mM	Sigma	X	X
[Leu15]-Gastrin I human	100uM	Sigma	X	X
Y-27632	10uM	Reagents Direct	X	X
Murine EGF	50ng/mL	Peprotech	X	X
Human R-Spondin2	100ng/mL	R&D	X	X
Noggin	100ng/mL	Peprotech	X	X
A 83-01	500nM	Tocris	X	X
Human Wnt-3a	100ng/mL	R&D	X	
Nicotinamide	10mM	Sigma	X	
SB 202190	10uM	Sigma	X	
