# Supplementary material for: Distinct Human Stem Cell Populations in Small and Large Intestine
Source: PLoS One. 2015 Mar 9;10(3):e0118792. doi: 10.1371/journal.pone.0118792 (PMC4353627; doi:10.1371/journal.pone.0118792)
Supplement: S5 Table — (PDF) [file pone.0118792.s010.pdf]

**S5 Table**

| Antibody | Manufacturereer | Clone | Conentration |
|---|---|---|---|
| CHGA | Millipore | LK2H10 | 1:400 |
| EPCAM | Abcam | E144 | 1:50 |
| MUC2 | Santa Cruz | Ccp58 | 1:150 |
| VIL1 | AbD SeroTec | ID2C3 | 1:100 |
| Alexa Fluor 488 secondary (anti-Rb) | Life Technologies | n/a | 1:300 |
| Alexa Fluor 594 secondary (anti-Ms) | Life Technologies | n/a | 1:300 |
