# Supplementary material for: Distinct Human Stem Cell Populations in Small and Large Intestine
Source: PLoS One. 2015 Mar 9;10(3):e0118792. doi: 10.1371/journal.pone.0118792 (PMC4353627; doi:10.1371/journal.pone.0118792)
Supplement: S6 Table — (PDF) [file pone.0118792.s011.pdf]

S6 Table

Target	Sequence
CHGA-Fwd	CGCTGTCCTGGCTCTTCTG
CHGA-Rev	CCTTTATTCATAGGGCTGTTCACA
EPHB2-Fwd	CCCAGTACACCTTCGAGATCCA
EPHB2-Rev	GGCGAACTGAGGCGAGAA
LGR5-Fwd	CCTGCGTCTGGATGCTAACC
LGR5-Rev	GGAATGCAGGCCACTGAAA
MYC-Fwd	CGTCTCCACACATCAGCACAA
MYC-Rev	TCTTGGCAGCAGGATAGTCCTT
MUC2-Fwd	TGGCTGGATTCTGGAAAACC
MUC2-Rev	TGGCTCTGCAAGAGATGTTAGC
ATOH1-Fwd	GCAATGTTATCCCGTCGTTCA
ATOH1-Rev	CCATCTGCAGGGTCTCATATTTG
GAPDH-Fwd	GGCATCCTGGGCTACACTGA
GAPDH-Rev	GGAGTGGGTGTCGCTGTTG
CPS1-Fwd	TCATGATGTTTGTGTATTGCTTCTTT
CPS1-Rev	AACAATATTCCTGGATTAGCCAAAT
MMP7-Fwd	CCAAATCAACCATAGGTCCAAGA
MMP7-Rev	CAGCACGGTGAGTCGCATA
RARRES1-Fwd	AGCGCATGCACACTGAACTC
RARRES1-Rev	GGGCTGAAACCCTGAGGAA
SPP1-Fwd	GCCAGTTGCAGCCTTCTCA
SPP1-Rev	AAAGCAAATCACTGCAATTCTCA
