# Supplementary material for: Distinct Human Stem Cell Populations in Small and Large Intestine
Source: PLoS One. 2015 Mar 9;10(3):e0118792. doi: 10.1371/journal.pone.0118792 (PMC4353627; doi:10.1371/journal.pone.0118792)
Supplement: S1 Methods — (DOCX) [file pone.0118792.s012.docx]

**S1 Methods:**

*Xenograft tumor analysis:* Rag-2/γc^-/-^ mice were anesthetized using isoflurane and transplanted with cells expanded from SI or LI (passage 5-11 on feeder cells) and colon CSCs (passage 14 on feeder cells). Cells were mixed 1:1 with HBSS/Matrigel and 0.5-1.0e6 cells were injected into the subcutaneous space. 1-4 months later, the mice were sacrificed via carbon dioxide and opened to observe tumor formation.

*Tumor cell culture:* Colon CSCs, originally obtained from tumors surgically removed from the liver of patients with metastatic colon cancer, were maintained in the feeder cell system as previously reported [1]. For purposes of this study, colon CSCs were also supplemented with R-Spondin 2 to allow for direct comparison to normal intestinal cells in xenograft experiments.

*Paraffin embedding:* Organoids were treated with 4% PFA (Sigma-Aldrich, MO) for 20 min at 4°C, rinsed in PBS and embedded in 5% gelatin (Sigma-Aldrich, MO) in PBS and solidified on ice for 20 min. The gelatin was fixed in 4% PFA for 1 hour at 4°C, rinsed in PBS and submitted for paraffin embedding. Freshly isolated tissue was fixed in 4% PFA for 2 hours at 4°C, rinsed in PBS and submitted for paraffin embedding.

*Immunofluorescence:* Antibodies against EPCAM, Chromogranin A (CHGA), Mucin 2 (MUC2) and Villin (VIL1) were diluted in PBS containing 0.5% Bovine Serum Albumin (BSA) (Fisher, PA) (S5 Table). Paraffin sections were deparaffinized and rehydrated, permeabilized with 0.1% Triton X100 (Sigma-Aldrich, MO) for 10 min, blocked with 5% BSA for 30 min, stained with primary antibodies for one hour followed by either anti-mouse or anti-rabbit Alexa Fluor secondaries (Life Technologies, NY) for 30 minutes. Images were taken using an Olympus IX71 Inverted microscope (Olympus, PA) with an Olympus DP71 camera (Olympus) and CellSens Standard Digital Imaging Software (Olympus).

*RNA isolation and Real-Time PCR:* Organoids grown in Matrigel were lysed and RNA isolated using the RNeasy kit (Qiagen, CA). RNA was quantified with the Nanodrop 2000c Spectrophotometer (Thermo Scientific, IL). Reverse transcription was performed using iScript RT Supermix (Bio-Rad, CA) followed by Real-Time PCR with SsoADV SYBR Green (Bio-Rad) on an ABI StepOne Plus Real-Time PCR machine (Applied Biosystems, CA). Primers were designed using Primer Express software (Applied Biosystems, CA) and purchased from Life Technologies (NY) (S6 Table). Expression was normalized to *GAPDH*. Error bars represent upper and lower error limits based on replicate variability. Statistical analysis was performed using Student’s t-test (*P*<0.05 was considered significant).

*mRNA Expression Assays: Eukaryotic Target Preparation, Hybridization and Data Processing:* Total RNA was isolated using the Qiagen miRNeasy kit (Qiagen, CA) according to the manufacturer’s protocol. Only RNA of highest quality and integrity was subjected to further processing after purification as defined by an absorption ratio 260/280>1.8 obtained by spectrophotometry on the NanoDrop 1000 (Thermo Scientific, IL) and a RIN value >8.0 via electrophoretic analysis on the Bioanalyzer 2100 (Agilent Technologies, CA). *In vitro* transcription (IVT) was performed using the Ambion MessageAmp Premier Enhanced assay protocol (Ambion, TX) starting with 500ng of purified total RNA. Confirmation of cRNA diversity was obtained using the Bioanalyzer 2100 to generate an electrophoretogram for the products of each IVT reaction regarding sample yield, integrity, and size diversity against transcripts derived from a Universal Human Reference RNA (Stratagene, CA). Fifteen micrograms of purified, amplified, biotin labeled cRNA were fragmented and hybridized onto Affymetrix Human Genome HGU133A 2.0 arrays (Affymetrix Corp. CA) for 18 hours. Washing, staining and scanning of arrays was performed on the Affymetrix Fluidics Station 450 and Scanner 3000 immediately after completion of hybridization according to the manufacturer’s protocol.

Microarray data were processed using the Robust Multi-array Average by importing raw CEL files into Partek software (Partek Genomics Suite, MO) resulting in RMA background correction, log base 2 transformation, quantile normalization and median polish probe set summarization. A 2 way ANOVA was performed across the three groups (LI, SI, feeders) versus gene expression and post-hoc testing was performed using the Students T-test to identify transcripts that were significantly changed between groups. It is important to note that feeder cells were also subjected to gene array analysis (n=5) to ensure they were profoundly different from the expanded SI and LI cells. Indeed, most of the 22,000 probe sets per array were significantly different when comparing expanded SI (20,830) and LI (19,791) cells to feeder cells. This gives us confidence that the expanded SI and LI cells are phenotypically very different from the feeder cells and that the feeder cells likely represent a constant background of gene expression in both cases. A q-value correction adjusted for false discovery rate (FDR) was established (q<0.05) to control for Type 1 errors arising from multiple tests.

1. Odoux C, Fohrer H, Hoppo T, Guzik L, Stolz DB, et al. (2008) A stochastic model for cancer stem cell origin in metastatic colon cancer. Cancer Res 68: 6932-6941.
